# Investigation of the Abundance of *Escherichia coli* and *Staphylococcus aureus* (Including Virulence Gene Profiles) and Heavy Metal Contamination in Camel Milk

**DOI:** 10.1002/vms3.70632

**Published:** 2025-09-30

**Authors:** Elahe Yazdanian Ghahfarokhi, Amir Shakerian, Reza Sharafati Chaleshtori, Ebrahim Rahimi

**Affiliations:** ^1^ Department of Food Hygiene ShK.C., Islamic Azad University Shahrekord Iran; ^2^ Research Center of Nutrition and Organic Products (R.C.N.O.P) ShK.C., Islamic Azad University Shahrekord Iran; ^3^ Research Center for Biochemistry and Nutrition in Metabolic Diseases Kashan University of Medical Sciences Kashan Iran; ^4^ Department of Veterinary ShK.C., Islamic Azad University Shahrekord Iran

**Keywords:** camel milk, *Escherichia coli*, heavy metal, *Staphylococcus aureus*, virulence genes

## Abstract

Camel milk (CM) may include foodborne pathogens, including *Staphylococcus aureus* and *Escherichia coli*, posing a health risk to consumers. The presence of heavy metals, including lead (Pb), cadmium (Cd) and arsenic (As), in CM may pose adverse health effects. This research attempted to assess the frequency of *S. aureus* and *E. coli*, together with their virulence characteristics, and to quantify the quantity of heavy metals in CM. In this research, 115 raw CM samples were collected from different rural areas of Chaharmahal Bakhtiari province of Iran. *E. coli* and *S. aureus* isolates were determined using a combination of traditional biochemical techniques and PCR. Antimicrobial susceptibility testing was performed using a panel of antibiotics from different classes. The identification of virulence genes in the isolated strains of *E. coli* and *S. aureus* was conducted by multiplex PCR. The levels of heavy metals in milk specimens were quantified using an atomic absorption spectrophotometer. *E. coli* was identified in 19.13% of the samples, whereas *S. aureus* was present in 23.47%. *S. aureus* isolates showed high resistance to penicillin (95.59%), tetracycline (TE) (81.48%) and clindamycin (59.29%). Moreover, *E. coli* strains showed high resistance to TE (90.90%), amikacin (81.81%), enrofloxacin (77.27%) and gentamicin (59.09%). *S. aureus* isolates frequently harboured *sea*, *nuc*, *tst* and *pvl* genes. In *E. coli* isolates, *eaeA* and *bfp* genes, indicative of enteropathogenic *E. coli* (EPEC), were most common. The average concentrations of heavy metals Cd, As and Pb in this investigation ranged from 0.0020 to 0.0053 ppm. This study found *E. coli* and *S. aureus* in some CM samples, emphasizing the need for improved hygiene. Although heavy metals were detected, their levels were generally low, requiring ongoing monitoring for consumer safety.

## Graphical abstract

GRAPHICAL ABSTRACT

Camel milk exhibited *Staphylococcus aureus* and *Escherichia coli* contamination with associated antibiotic resistance and virulence genes. Concurrently, heavy metal levels (Pb, As and Cd) in these samples were low and below safety limits, providing a comprehensive assessment of dual hazards in this vital food source.

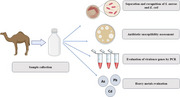

**Abbreviations**
As: arsenic
BFP: bundle‐forming pili
Cd: cadmium
CLSI: Clinical Laboratory Standards Institute
CM: camel milk
*E. coli*
: *Escherichia coli*
EMB: eosin methylene blue agar
EPEC: enteropathogenic *E. coli*
ETEC: enterotoxigenic *E. coli*
LIA: lysine iron agar
LOD: limit of detection
LT: heat‐labile toxin
MAC: maximum allowable concentration
MCA: MacConkey agar
MHA: Mueller–Hinton agar
Pb: lead
PVL: Panton–Valentine leukocidin
*S. aureus*
: *Staphylococcus aureus*
SEs: staphylococcal enterotoxins
STa: heat‐stable toxin
STEC: Shiga toxin‐producing *E. coli*
TSI: triple sugar iron agar
TSST‐1: toxic shock syndrome toxin‐1
WHO: World Health Organization

## 1 Introduction

Milk is a diverse and nutritious product that has significant effects on people's lives across the world (Xiang et al. 2021). It has a high concentration of key minerals, such as calcium, protein, vitamin D and potassium, making it an important part of a balanced diet (Moatsou and Sakkas 2019). Camels are essential to the livelihoods of several people, especially in the Middle East and Africa (Lezzoum‐Atek et al. 2023). Camel milk (CM) is a primary dietary factor in arid and semi‐arid regions, when feed supplies are limited. Recent findings regarding its chemical composition and health advantages have resulted in a substantial rise in its popularity and commercial development (Tasse et al. 2022). CM has superior nutritional content and therapeutic benefits relative to the milk of other animals. The individual components of CM differ markedly from those of goat, sheep and cow milk (Abera et al. 2016). In comparison to bovine milk, CM has elevated levels of vitamin C and niacin, along with a significant quantity of water, especially throughout the arid season when camels experience dehydration (Almasri et al. 2024). Moreover, CM is a crucial component of the nomadic diet owing to its therapeutic benefits for various ailments and its superior concentration of vitamins, minerals, immunoglobulins and antioxidants relative to the milk of other kinds of mammals (Ali et al. 2024).

Currently, foodborne bacteria are considered a primary cause of sickness outbreaks linked to the consumption of contaminated food (Javed et al. 2024). The World Health Organization (WHO) estimates that foodborne infections cause around 600 million cases and 420,000 fatalities worldwide annually (Pires and Devleesschauwer 2021). Milk serves as an exceptional culture medium for microbial development. Raw camel's milk may have pathogenic microorganisms for humans, with contamination often originating from three primary places (Sabir et al. 2024; Yang et al., 2025). Moreover, the improper administration of antibiotics is a prevalent issue in both human and veterinary medicine, potentially leading to the emergence of multidrug‐resistant microbes. *Staphylococcus aureus* is a significant contributor to foodborne diseases globally, owing to its capacity to manufacture several heat‐stable enterotoxins. *S. aureus* is a significant pathogen in raw milk and the primary pathogen associated with mastitis globally. Infectious diseases induced by *S. aureus* are linked to the expression of virulence‐related factors (Javed et al. 2025). *S. aureus* employs several virulence factors, such as enzymes and exotoxins, which play a crucial role in the onset and advancement of infections. *S. aureus* may possess many virulence genes that encode enterotoxins, enterotoxin‐like exfoliative toxin, toxic shock syndrome toxin‐1 (TSST‐1) and Panton–Valentine leukocidin (PVL) (Alghizzi and Shami 2021). *Escherichia coli* is one of the major bacteria responsible for foodborne illnesses, which negatively impacts human health. This bacterium is a Gram‐negative bacillus, classified under the Enterobacteriaceae family (Saeed et al. 2022). *E. coli* is a predominant occupant of the digestive system in most kinds of mammals, such as humans (Muthukumaran et al., 2023). Although the majority of *E. coli* species are benign, several strains are recognized as dangerous, leading to serious intestinal and extraintestinal illnesses in people (Geletu et al. 2022). Raw milk has a greater likelihood of contamination with *E. coli*, thereby posing a significant risk to public health.

Heavy metals are closely linked to health issues in humans among many pollutants (Kerdoun and Djafer 2024). Augmented industrial and agricultural activity resulted in elevated concentrations of metals, including iron, manganese, zinc, copper, aluminium, lead, chromium, mercury and nickel, in the water, air and soil (Abba et al. 2021). Animals can ingest contaminated food and water, or graze in polluted areas, which can lead to the accumulation of dangerous heavy metals like Cd, Pb and As in their milk (Parsaei et al. 2019). Chronic and acute exposure to heavy metals can lead to a range of serious health problems, including various cancers, tumours, autoimmune diseases, oxidative stress, enzyme dysfunction, iron deficiency and damage to the liver and kidneys (Mostafidi et al. 2016, Rehman et al. 2018). Assessing the concentrations of Pb, Cd and As in milk is essential for public health, because milk is a fundamental food item ingested by individuals of all ages, particularly newborns and young children who are most susceptible to the harmful impacts of heavy metals (Yasotha et al. 2021). CM provides important nutritional and health benefits. However, consuming it poses serious health risks because of common microbial contamination by pathogens like *S. aureus* and *E. coli*. These pathogens often carry antibiotic resistance genes, and there can also be harmful heavy metals present. As there is limited information on these safety concerns in Iranian CM, this research was necessary to evaluate contamination levels and the related risks. This will help guide public health and food safety measures. This research attempted to identify the presence of *S. aureus* and *E. coli* in raw CM sourced from Chaharmahal Bakhtiari province, Iran. Furthermore, it examined the extent of antibiotic resistance and the existence of virulence‐related genes in these bacteria to comprehend the dissemination of antimicrobial resistance in the area. This research also investigated the concentrations of heavy metals in CM sourced from Iran.

## 2 Materials and Methods

### 2.1 Sample Collection and Geographical Location of the Study

This research examined 115 raw milk samples, one from each of 115 camels, gathered from diverse rural regions of Chaharmahal Bakhtiari province in Iran from April to July 2023. This study was executed in compliance with ethical guidelines and national regulations governing medical research (ethical code: IR.IAU.SHK.REC.1403.341). The camels were chosen at random, mirroring the irregular breeding patterns characteristic of this semi‐arid, nomadic area. Their food mostly comprises the grasses found in their native habitat. Samples were promptly transported under refrigerated conditions to the laboratory for bacterial separation and characterization.

### 2.2 Separation and Recognition of *S. aureus* and *E. coli* Bacteria

To isolate and determine the strains of *E. coli* and *S. aureus*, milk samples were inoculated onto sheep blood agar (Condalab, Spain) and MacConkey agar (MCA) (Ibresco, Iran). In summary, 100 µL of milk specimens were spread onto 5% sheep blood agar and MCA. Distinct colonies that appeared to be *Staphylococcus* species were first taken from blood agar and then moved to Mannitol Salt Agar (MSA, Condalab, Spain). On MSA, colonies that showed a yellow colour or a yellow halo indicated mannitol fermentation. This fermentation changed the pH and turned the agar from pink to yellow, suggesting they were *S. aureus*. In contrast, colonies that stayed pink but continued to grow were seen as other *Staphylococcus* species. All suspected isolates were then tested further with confirmatory biochemical tests. Pink colonies suspected to be *E. coli* were isolated from MCA and then grown on a second type of agar called eosin methylene blue agar (EMB) (Ibresco, Iran). Colonies with a specific appearance on this second agar—a green metallic sheen with a black centre—confirmed the presence of *E. coli*. The collected specimens were incubated under aerobic conditions at 37°C for 24 h. To further validate the isolated strains, biochemical assays were conducted, including motility, oxidative/fermentative glucose destruction, citrate utilization, urease generation, indole fermentation, tryptophan degradation, glucose degradation (methyl red test) and growth on triple sugar iron agar (TSI) and lysine iron agar (LIA) (Ijaz, Sabiret al. 2024).

### 2.3 Antibiotic Susceptibility Assessment

In accordance with Clinical Laboratory Standards Institute (CLSI) guidelines, an antibiotic susceptibility test was performed with the Kirby Bauer disc diffusion technique on Mueller–Hinton agar (MHA) (Merck, Germany) (Ranjbar et al. 2018). The sensitivity of *S. aureus* strains was evaluated through various classes of antibiotics: azithromycin (AZM; 15 µg/disc), penicillin (P; 10 U/disc), levofloxacin (LVX; 5 µg/disc), tetracycline (TE; 30 µg/disc), gentamicin (GM; 10 µg/disc), oxacillin (OXA; 30 µg/disc), erythromycin (E; 15 µg/disc), clindamycin (CLI; 2 µg/disc), chloramphenicol (C; 30 µg/disc) and vancomycin (V; 30 µg/disc). The susceptibility of *E. coli* strains against 12 different antibiotics was evaluated: TE (30 U/disc), ampicillin (AM; 10 U/disc), cefotaxime (CTX; 30 µg/disc), GM (10 µg on disc), ciprofloxacin (CIP; 5 µg/disc), amikacin (AN; 30 U/disc), ceftazidime (CAZ; 30 µg/disc), imipenem (IPM; 30 U/disc), enrofloxacin (ENR; 5 µg/disc), trimethoprim‐sulfamethoxazole (SXT; 1.25/23.75 µg/disc), meropenem (MEM; 10 µg/disc) and chloramphenicol (C; 30 µg/disc). All antibiotics were obtained from PadtanTeb (Iran) Company. *E. coli* ATCC 25922 and *S. aureus* ATCC 25923, which were purchased from the National Center for Genetic and Biological Resources of Iran, were used as reference strains. The CLSI zone diameter breakpoints are utilized to assess the antibacterial vulnerability of the examined isolates.

### 2.4 DNA Extraction and PCR Confirmation

The DNall Plus DNA extraction kit (ROJE, Iran) was employed to separate genomic DNA from *S. aureus* and *E. coli* strains in accordance with the manufacturer's guidelines. The quality and quantity of isolated genomic DNA were evaluated utilizing 1% agarose gel and a Nanodrop 2000 spectrophotometer (Thermo Scientific, USA). The identification of organisms within the genera *S. aureus* and *E. coli* was achieved by the specialized *16S rRNA* PCR technique, which amplifies the *16S rRNA* gene sequences unique to these genera. The primer sequences applied are presented in Table 1. A 25‐µL PCR reaction mixture was prepared, involving 12.5 µL of Taq DNA Polymerase Master Mix RED (Ampliqon, Denmark), 0.5 µM of each primer and 1 µL of template DNA, and was conducted in a Coyote Thermal Cycler (China). The thermal program was performed in the following order: 10 min at 95°C for one cycle, followed by 30 s at 95°C, 45 s at 58°C and 30 s at 72°C for 35 cycles. The PCR result was evaluated by 1% gel electrophoresis (Ijaz, Ghummanet al. 2024).

**TABLE 1:** List of target genes and oligonucleotide primers employed in the PCR reactions conducted in this investigation.

Strain	Gene name	Sequence (5′–3′)	Product size	*T*_m_ (°C)	References
*Staphylococcus aureus* virulence genes	*Nuc*	F: GCGATTGATGGTGATACGGTT R: AGCCAAGCCTTGACGAACTAAAGC	279	58	Karimzadeh and Ghassab (2022)
	*sea*	F: GGTTATCAATGTGCGGGTGG R: CGGCACTTTTTTCTCTTCGG	102	59	Akindolire et al. (2015)
	*seb*	F: GTATGGTGGTGTAACTGAGC R: CCAAATAGTGACGAGTTAGG	164	56	Sharma et al. (2017)
	*sec*	F: AGATGAAGTAGTTGATGTGTATGG R: CACACTTTTAGAATCAACCG	451	57	Sharma et al. (2017)
	*sed*	F: CCAATAATAGGAGAAAATAAAAG R: ATTGGTATTTTTTTTCGTTC	278	58.5	Sharma et al. (2017)
	*tst*	F: ACCCCTGTTCCCTTATCATC R: TTTTCAGTATTTGTAACGCC	326	59	Bayrakal and Aydin (2024)
	*Pvl*	F: ATCATTAGGTAAAATGTCTGGACATGATCCA R: GCATCAACTGTATTGGATAGCAAAAGC	433	60	Sadat et al. (2022)
	*16S rRNA*	F: GTAGGTGGCAAGCGTTACC R: CGCACATCAGCGTCAG	228	56	Akindolire et al. (2015)
*Escherichia coli* virulence genes	*stx1*	F: GAAGAGTCCGTGGGATTACG R: AGCGATGCAGCTATTAATAA	130	60	Abdlla et al. (2024)
	*stx2*	F: ACCGTTTTTCAGATTTTGACACATA R: TACACAGGAGCAGTTTCAGACAGT	298	59	Mohammadi et al. (2013)
	*eaeA*	F: CACACGAATAAACTGACTAAAATG R: AAAAACGCTGACCCGCACCTAAAT	376	58.5	Mohammadi et al. (2013)
	*estA*	F: TCCCCTCTTTTAGTCAGTCAACTG R: TCCCCTCTTTTAGTCAGTCAACTG	163	57	Liu et al. (2021)
	*hlyA*	F: ACCGCTGGCAACAAAGGATA R: AACAAGGATAAGCACTGTTCTGGCT	1177	60	Younis et al. (2021)
	*elt*	F: TCTCTATGTGCATACGGAGC R: CCATACTGATTGCCGCAAT	322	57.5	Liu et al. (2021)
	*bfp*	F: AATGGTGCTTGCGCTTGCTGC R: GCCGCTTTATCCAACCTGGTA	336	61	Zarei Ahmady et al. (2023)
	*16S rRNA*	F: GTTAATACCTTTGCTCATTGA R: ACCAGGGTATCTAATCCTGTT	340	57	Liu et al. (2021)

### 2.5 Evaluation of Virulence Genes

This work evaluated the existence of enterotoxin genes *sea*, *seb*, *sec*, *sed*, *nuc* and TSST‐1 (tst) in *S. aureus* isolates obtained from CM using the multiplex PCR technique. Seven virulence genes associated with diarrheagenic *E. coli* were identified using the PCR method: *stx1* and *stx2* for Shiga toxin‐producing *E. coli* (STEC), *estA* and *elt* for enterotoxigenic *E. coli* (ETEC), *hylA* (alpha‐hemolysin), *bfp* and *eaeA* for enteropathogenic *E. coli* (EPEC). All specific primers are included in Table 1. The PCR for amplifying the target gene was performed in a total reaction volume of 25 µL. Each reaction mixture included 12 µL of Master Mix RED 2x (Ampliqon, Denmark), 5 µL of template DNA, 1 µL of each 10 µM primer and 7 µL of DEPC‐treated water. The thermal cycling protocol began with an initial denaturation step at 95°C for 10 min. This was followed by 30 amplification cycles, each containing denaturation at 95°C for 30 s, annealing at a specific optimized temperature (Table 1) for 30 s and extension at 72°C for 30 s. The extension time was adjusted as needed based on the expected size of the target gene amplicon. Following amplification, the PCR products were resolved using electrophoresis on a 1% agarose gel, which was then dyed with a safe DNA‐intercalating dye for viewing under ultraviolet light.

### 2.6 Heavy Metals Evaluation

Heavy metal levels of As, Pb and Cd were measured in 49 randomly chosen raw CM samples from a total of 115 collected. This random sampling made sure the heavy metal data represented the overall milk samples and avoided any bias from bacterial contamination. Measurements were taken using an atomic absorption spectrophotometer (Shimadzu‐AA‐670, Japan). For Pb and Cd, specific cathode lamps with wavelengths of 288.3 and 288.8 nm were used. The instrument was carefully calibrated for each element using certified standard solutions of Pb and Cd. From these, a standard curve was created to ensure precise measurement. As concentrations in the milk samples were measured with the same spectrophotometer at a wavelength of 193.7 nm. The limit of detection (LOD) represents the lowest concentration that can be reliably detected and measured for each element in the samples. It was determined to be 0.000698 ppm for As, 0.000665 ppm for Cd and 0.00342 ppm for Pb. These LODs ensure that all reported concentrations were within the measurable range of the instrument.

### 2.7 Statistical Examination

Statistical evaluations were conducted with GraphPad PRISM software (version 10.4.1) to examine variations in prevalence, antibiotic resistance and distribution of virulence or enterotoxin‐producing genes. The chi‐square test and Fisher's exact test were used to evaluate differences in the prevalence of *S. aureus* and *E. coli*. They compared antibiotic resistance rates across different drug classes and examined the distribution patterns of specific virulence genes among isolates. These comparisons provided valuable insights into bacterial traits and their impact on public health (*p* < 0.05).

## 3 Results

### 3.1 Evaluation of Distribution of *E. coli* and *S. aureus* Isolates in CM

Over a period of 5 months, 115 specimens of CM were gathered from various rural and nomadic regions of Chaharmahal Bakhtiari province. The findings from standard biochemical assays indicated that the frequency of *E. coli* in 115 samples was 19.13% (22/115), whereas the frequency of *S. aureus* was 23.47% (27/115) (Table 2). Furthermore, all of *E. coli* and *S. aureus* isolates detected in CM samples were validated using particular primers for the *16S rRNA* gene.

**TABLE 2:** The results of frequency and antibiotic resistance pattern of *Escherichia coli* and *Staphylococcus aureus* strains obtained from camel milk.

					Phenotype resistance rate (%)					
					R		S		I	
Strain	Sample size	Confirmed *S. aureus*	Antibiotic	Antibiotic class	No. of isolates	%	No. of isolates	%	No. of isolates	%
*S. aureus*	115	27/115 (23.47%)	AZM	Macrolide	8/27	29.62	19/27	70.37	0/27	—
			P	Beta‐lactam	25/27	95.59	0/27	—	2/27	7.40
			LVX	Fluoroquinolone	12/27	44.44	15/27	55.55	0/27	—
			TE	Tetracycline	22/27	81.48	3/27	11.11	2/27	7.40
			GM	Aminoglycoside	14/27	51.85	13/27	48.14	0/27	—
			OXA	Beta‐lactam	7/27	25.92	11/27	40.74	9/27	33.33
			E	Macrolide	9/27	33.33	18/27	66.66	0/27	—
			CLI	Lincomycin	16/27	59.25	8/27	29.62	3/27	11.11
			C	Phenicol	4/27	14.81	23/27	85.18	0/27	—
			V	Glycopeptide	7/27	25.92	16/27	59.25	4/27	14.81
*E. coli*	115	22/115 (19.13%)	TE	Tetracycline	20/22	90.90	2/22	9.09	0/22	—
			AM	Beta‐lactam	18/22	81.81	4/22	18.18	0/22	—
			CTX	Cephalosporin	7/22	31.81	12/22	54.54	3/22	13.63
			GM	Aminoglycoside	13/22	59.09	9/22	40.90	0/22	—
			CIP	Quinolone	2/22	9.09	20/22	90.90	0/22	—
			AN	Aminoglycoside	6/22	27.27	13/22	59.09	3/22	13.63
			CAZ	Cephalosporin	5/22	22.72	17/22	77.27	0/22	—
			IPM	Carbapenem	2/22	9.09	20/22	90.90	0/22	—
			ENR	Fluoroquinolone	17/22	77.27	5/22	22.72	0/22	—
			SXT	Sulfonamide	4/22	18.18	16/22	72.72	2/22	9.09
			MEM	Beta‐lactam	1/22	4.54	17/22	77.27	4/22	18.18
			C	Phenicol	8/22	36.36	14/22	63.63	0/22	—

Abbreviations: AM, ampicillin; AN, amikacin; AZM, azithromycin; CAZ, ceftazidime; CIP, ciprofloxacin; CLI, clindamycin; CTX, cefotaxime; ENR, enrofloxacin; GM, gentamicin; I, intermediate; IPM, imipenem; LVX, levofloxacin; MEM, meropenem; OXA, oxacillin; R, resistance; S, sensitive; SXT, trimethoprim‐sulfamethoxazole; TE, tetracycline.

### 3.2 Evaluation of the Antibiotic Resistance Profile

The antibiotic susceptibility profile of *S. aureus* strains obtained from raw CM was assessed using nine distinct classes of antibiotics, involving beta‐lactams, macrolides, fluoroquinolone, aminoglycosides, TEs, lincosamides, glycopeptide, cephalosporin and phenicols (Table 2). This study revealed that the highest rates of antibiotic resistance in *S. aureus* isolates are associated with penicillin (95.59%), TE (81.48%) and CLI (59.29%). In other words, the highest amount of antibiotic resistance among *S. aureus* isolates obtained from CM was related to beta‐lactams, TEs and lincosamides. In addition, 44.44% and 51.85% of *S. aureus* strains were observed compared to LVX and GM. After that, the highest antibiotic sensitivity of *S. aureus* strains in macrolide classes was reported for AZM and erythromycin, 70.37% and 66.66%, respectively. In addition, 85% of *S. aureus* strains were sensitive to chloramphenicol. Further studies showed that *S. aureus* strains isolated from CM have moderate sensitivity to vancomycin (59.25%), GM (48.14%) and OXA (40.74%). To evaluate the microbiological sensitivity profile of *E. coli* isolates obtained from CM, nine distinct classes of antibiotics were employed: beta‐lactam, aminoglycoside, macrolide, cephalosporin, quinolone, carbapenem, fluoroquinolone, sulfonamide, phenicol and TE (Table 2). The *E. coli* strains obtained from CM exhibited the greatest resistance levels to TE, beta‐lactams, fluoroquinolones and aminoglycosides. The greatest levels of antimicrobial resistance in *E. coli* strains were seen for TE (90.90%), AN (81.81%), ENR (77.27%) and GM (59.09%). *E. coli* bacteria derived from camel's milk had the greatest susceptibility to IPM (90.90%), CIP (90.90%), MEM (77.27%), SXT (72.72%) and chloramphenicol (63.63%) antibiotics, respectively. Further information is shown in Table 2.

### 3.3 Assessment of Virulence Genes With the Multiplex PCR Technique

**FIGURE 1:**
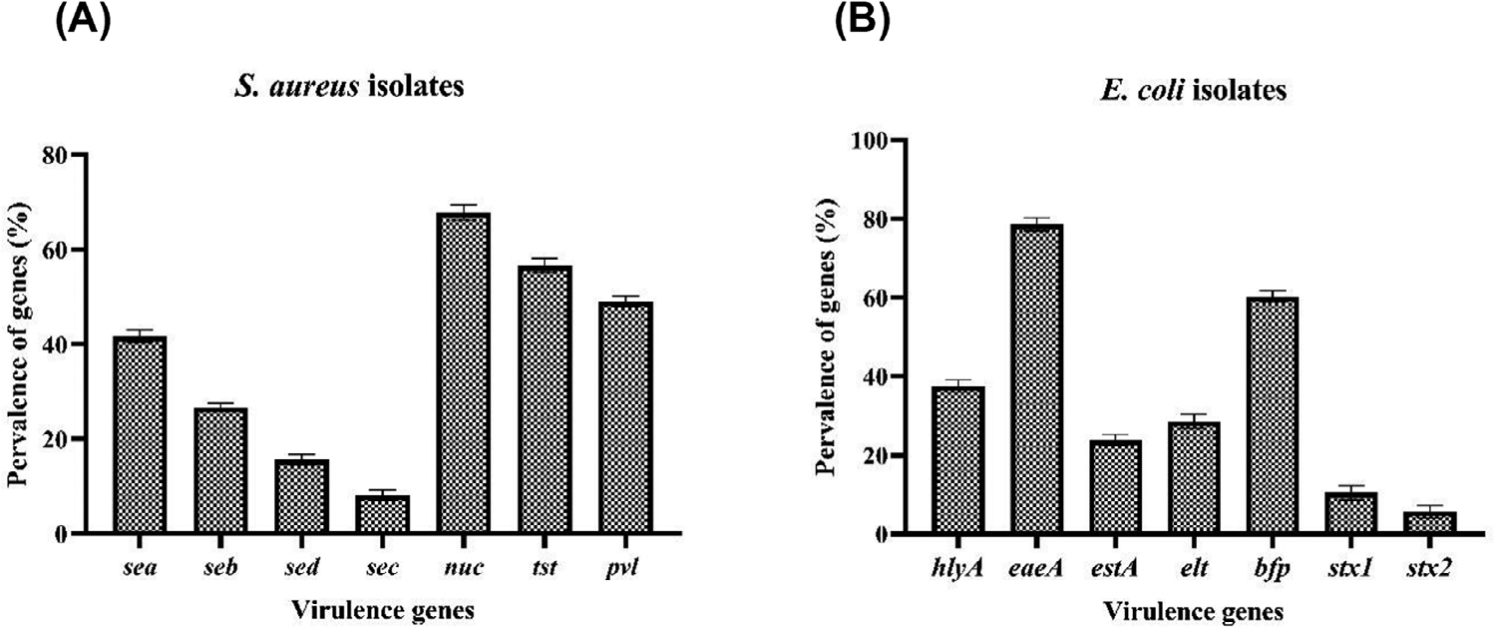
The findings of the examination of the prevalence of virulence genes in *Staphylococcus aureus* (A) and *Escherichia coli* (B) isolates derived from camel milk samples.

The PCR analysis of pure DNA revealed the presence of four major toxin genes in isolated *S. aureus*: *sea*, *seb*, *sec* and *sed* (Figure 1A). The predominant genotype was *sea* (40.74%, 11/27), which was followed by *seb* (25.95%, 7/27), *sed* (14.81%, 4/27) and *sec* (7.40%, 2/27). The findings of this investigation indicated that 66.66% (18/27) of *S. aureus* isolates derived from CM were positive for the *nuc* gene. The *tst* gene in *S. aureus*, responsible for encoding TSST‐1, was identified in 55.55% (15/27) of the *S. aureus* isolates recovered from CM in this investigation. The *pvl* gene was found in 27 isolates of *S. aureus*. Thirteen of these isolates (48.15%) were found to possess the *pvl* gene (Figure 1A). PCR investigation with *16S rRNA* primers validated the detection of *E. coli* DNA in 22 samples. This step ensures that the isolates being studied are indeed *E. coli*. The investigation focused on the presence of seven critical virulence‐related genes (*hlyA*, *eaeA*, *estA*, *elt*, *bfp*, *stx1* and *stx2*) among these 22 verified *E. coli* isolates (Figure 1B). The *stx1* and *stx2* genes, which indicate the presence of STEC, were found in only 9.09% (2/22) and 4.54% (1/22) of the isolates, respectively. In this study, 36.36% (8/22) of *E. coli* isolates obtained from CM were positive for the *hlyA* gene. The *bfp* and *eaeA* genes, present in EPEC strains, were identified in 59.09% (13/22) and 77.27% (17/22) of the *E. coli* isolates in this investigation, respectively. Therefore, it can be concluded that the most serotype of *E. coli* found in this study is EPEC. *estA* and *elt* genes, virulence factors of ETEC strains, were identified in 22.72% (5/22) and 27.27% (6/22) of *E. coli* isolates, respectively.

### 3.4 Evaluation of Heavy Metals in CM

From the 115 CM samples collected over 5 months in Chaharmahal Bakhtiari province, *E. coli* was found in 19.13% (22/115) and *S. aureus* in 23.47% (27/115). All isolates were confirmed by *16S rRNA* gene PCR. Researchers then specifically analysed these 49 microbiologically positive samples to measure their concentrations of Pb, Cd and As. This analysis allowed for an assessment of heavy metal levels in milk that already had potential microbial safety concerns (Table 3). The lead concentration in the analysed milk samples containing *S. aureus* varied from 0.0010 to 0.0055 ppm, with a mean of 0.0053 ppm. The CM samples showed low levels of heavy metals. Cd was found in concentrations between 0.0031 and 0.0032 ppm. Pb levels ranged from 0.0039 to 0.0053 ppm. All these amounts were well below the maximum allowable concentrations (MACs). Arsenic exhibited the lowest quantity across all investigated milk types, namely, 0.0020 ± 0.001 ppm in 27 samples containing *S. aureus* and 0.0024 ± 0.001 ppm in 22 samples carrying *E. coli*. The concentrations of Cd and Pb in the analysed CM samples exceeded those of As and exhibited a considerable difference (*p *< 0.05).

**TABLE 3:** Concentrations of Pb, Cd and As in camel milk samples.

	Concentration of Pb (ppm)			Concentration of Cd (ppm)			Concentration of As (ppm)		
Samples	Mean	Max.	Min.	Mean	Max.	Min.	Mean	Max.	Min.
27 milk containing *Staphylococcus aureus*	0.0053 ± 0.011	0.055	0.0013	0.0031 ± 0.001	0.0059	0.0012	0.0020 ± 0.008	0.0041	0.001
22 milk containing *Escherichia coli*	0.0039 ± 0.006	0.034	0.001	0.0032 ± 0.002	0.0043	0.0015	0.0024 ± 0.001	0.0067	0.0012

*Note*: Data expressed as mean ± standard deviation (SD).

## 4 Discussion

Milk and dairy products have long been seen as essential for a nutritious diet. They provide energy, protein, vital minerals, vitamins, such as vitamin B2 (riboflavin) and, notably, calcium (Zhang et al. 2022). Research indicates that the use of milk and dairy products may promote muscle development and assist in managing health issues, such as hypertension, diabetes, cancer and obesity (Chauhan et al. 2021). The existence of harmful microbes in these foods poses a considerable threat to food quality and human health (Bastam et al. 2021). Moreover, the presence of heavy metals in milk endangers consumer health and undermines the image of the dairy sector, adversely affecting customer trust and possibly resulting in economic losses for farmers and producers (Yan et al. 2022). The objective of this research was to examine the incidence of *E. coli* and *S. aureus* isolates in CM samples obtained from rural and nomadic regions of Chaharmahal Bakhtiari province. This research examined the incidence of enterotoxigenic genes in isolated strains. Moreover, the concentration of heavy metals Cd, Pb and As in the collected milk samples was evaluated. The investigation on the occurrence of *S. aureus* in unpasteurized CM indicates that out of 115 milk specimens, 27 (23.47%) tested positive for *S. aureus*, with all bacteria verified by PCR to possess the *16S rRNA* gene. The results of this study were consistent with the studies conducted in Egypt (38.5%) (Elhosseny et al. 2018) and Kenya (34.9%) (Gitao et al. 2014) on raw CM. However, the present study discovered a higher incidence than previous studies conducted in Ethiopia (Serda et al. 2018), Iran (Rahimi and Alian 2013) and Turkey (Aydin et al. 2011), which showed 11.45%, 11% and 10.2% of positive samples, respectively. Research has shown differing degrees of *S. aureus* infection in CM (Teshome et al. 2016). Research suggests a significant prevalence of these bacteria, raising concerns over possible food safety problems. Although CM may provide potential health benefits, it is crucial to ensure its safety and mitigate the risk of bacterial contamination for consumption. The incidence of *S. aureus* in diverse raw milk samples differs by country. In the study conducted in China, fresh buffalo milk specimens had the highest prevalence of *S. aureus* (36%), subsequent to goat milk (32%), CM (24%) and yak milk (20%) (Liu et al. 2022).

This research revealed the frequency of *E. coli* in CM specimens at 19.13%. *E. coli* was confirmed in 22 samples out of 115 milk samples collected by conventional biochemical methods and amplification of *16S rRNA* gene fragment. The presence of *E. coli* in raw dromedary CM poses a risk to human health (Bessalah et al. 2016). The findings of Saeed et al.’s (2022) research reveal that *E. coli* was detected in 24% (12/50) of the analysed fresh CM specimens and 8% (4/50) of the examined farm raw CM specimens via conventional biochemical methods. The overall prevalence of *E. coli* bacteria in raw milk and conventional dairy product samples from investigations done in Switzerland (Stephan et al. 2008), Iran (Mohammadi et al. 2013), Italy (Nobili et al. 2016), Egypt (Zeinhom and Abdel‐Latef 2014) and China (Xi et al. 2015) ranged from 1% to 27%, which is lower than the prevalence ratio we observed.

The extensive use of antibiotics in dairy production, for both illness treatment and growth promotion, substantially contributes to the emergence of antibiotic resistance. The regular or excessive use of antimicrobial drugs may result in the emergence of defence mechanisms in bacteria inside animals, diminishing their susceptibility to these treatments (Ijaz et al. 2023). These resistant bacteria may then contaminate milk and infiltrate the human food chain. Consumption of this milk may expose individuals to antibiotic‐resistant bacteria, hence increasing the chance of challenging illnesses (Lemma et al. 2021). Moreover, the existence of antibiotic residues in milk might negatively impact human health, especially in persons with compromised immune systems (Lemma et al. 2021). The antimicrobial resistance patterns of *S. aureus* indicate significant resistance to most widely used drugs. *S. aureus* isolates derived from CM samples exhibited 95.59% resistance to penicillin, 81.48% resistance to TE and limited resistance to CLI. *S. aureus* isolates were relatively sensitive to vancomycin, chloramphenicol, OXA and AZM. Research in Ethiopia revealed that *S. aureus* isolates exhibited significant resistance to AM, while demonstrating sensitivity to AN, gentamycin and TE (Girmay et al. 2020). Oliveira et al. (2011) demonstrated that all *S. aureus* strains isolated from cow's milk displayed resistance to both penicillin‐G and AM. A separate study indicated that *S. aureus* isolates derived from CM affected by mastitis exhibited significant resistance to piperacillin, tazobactam, sulfonamides and amoxicillin, attributed to the widespread and improper application of these administered antibiotics in veterinary medicine in Egypt (Ali et al. 2024). This study revealed that *E. coli* strains from CM demonstrated resistance to TE (90.90%), AM (81.81%) and ENR (77.27%), while showing susceptibility to chloramphenicol (63.63%), IPM (90.90%), MEM (77.27%), SXT (72.72%) and CIP (90.90%). The highest antibiotic resistance among *E. coli* and *S. aureus* isolates in this study was observed related to beta‐lactam and TE classes. In another study, 28 isolates of *E. coli* were identified in CM samples, whereas 5 isolates were identified in CM powder samples. *E. coli* strains exhibited absolute resistance to penicillin‐G (100%), followed by chloramphenicol (87.87%), amoxicillin (81.81%) and erythromycin (63.63%) (Dhuria et al. 2024). This resistance can arise from several factors, including poor hygiene practices by the milker, the health of the camels themselves, environmental contamination, inadequate sanitation of milking equipment and suboptimal storage and transportation conditions. The emergence of bacteria resistant to multiple drugs necessitates the implementation of strict measures to control its spread and addresses the growing challenge of antimicrobial resistance in food animals (Nato et al. 2019).

Staphylococcal enterotoxins (SEs) are a collection of toxins generated by the bacteria *S. aureus*. These toxins may infect milk and dairy products, resulting in a severe foodborne sickness known as staphylococcal food poisoning (Ishaq et al. 2022). *sea*, *seb*, *sec* and *sed* are genes within the *S. aureus* bacteria that code for the synthesis of various SEs. These genes direct the bacterial cell to produce the corresponding enterotoxin proteins (SEA, SEB, SEC, SED) (Fanelli et al. 2022; Ghauri et al. 2021). According to the results, the dominant genotype was *sea* (40.74%, 27/11), followed by *seb* (25.95%, 27/7), *sed* (14.81%, 27/4) and *sec* (7.40%, 2/27). Research indicates that the incidence of the *sea* gene in *S. aureus* collected from milk is generally greater than that of the *seb* gene, with investigations showing that *sea* is found in approximately 30%–50% of isolates (Rajabi et al. 2023). In the study conducted by Sadat et al. (2022), the *sea* gene was the most predominant gene detected in milk samples, following the *seb* and *sec* genes. The findings correspond with the discoveries of Chao et al. (2015), who noted a similar prevalence of enterotoxin genes in *S. aureus* isolates from China and Hungary; however, the *sea*, *seb* and *saw* genes were not observed. *S. aureus* bacteria with the PVL toxin are very perilous. These bacteria may induce severe illnesses in humans, including skin diseases such as recurrent boils and extensive dermatological issues, as well as a potentially fatal pulmonary infection known as necrotizing pneumonia (Chieffi et al. 2020). In this study, PVL‐positive *S. aureus* isolates were detected in 48.15% of all analysed *S. aureus* samples. In line with the current research, a study showed 11 isolates with *pvl* gene in CM (*n* = 4, 25%), camel nasal samples (*n* = 5, 31.3%) and human abscesses (*n* = 2, 100%) (Mahran et al. 2020). The *tst* gene was identified in 55.55% of isolates. This toxin induces toxic shock syndrome, characterized by elevated temperature, extensive itchy rash, skin desquamation, low blood pressure and dysfunction in three or more organ systems. Prior research found a *tst* gene frequency of 25.6%, in conjunction with different enterotoxigenic genes, in the milk of cows afflicted with mastitis, corroborating our results (Srinivasan et al. 2006).

STEC strains are pathogenic to humans and associated with haemorrhagic colitis and haemolytic uremic syndrome. Shiga toxins (Stx1 and Stx2) are the primary virulence factors of these bacteria (Tahamtan et al. 2010). In this investigation, the *stx2* gene was identified in a single isolate (4.54%), whereas the *stx1* gene was detected in 2 isolates out of 22 samples. EPEC has two essential virulence genes: *bfp* and *eaeA*. The *bfp* gene produces bundle‐forming pili (BFP), which mediate the first adhesion of EPEC to the intestinal epithelium and promote bacterial accumulation. The *eaeA* gene encodes intimin, an outer membrane protein crucial for the development of adhering and effacing (A/E) lesions. The coexistence of both *bfp* and *eaeA* genes is indicative of EPEC and substantially enhances its pathogenicity (Ram et al. 2024). The *eaeA* gene, responsible for encoding the intimin protein, was the most abundant gene in our analysis, accounting for 77.27% (17/22). Another important finding is that *bfp* was detected in 59.09% (13/22) of *E. coli* isolates, which is another characteristic of the EPEC group. This may suggest the existence of atypical EPEC variants in the nation, aligning with global results that aberrant EPEC variants are more prevalent than conventional ones. ETEC generates toxins that induce diarrhoea. The *elt* gene encodes the heat‐labile toxin (LT), whereas the *estA* gene produces the heat‐stable toxin (STa). These toxins disturb the fluid and electrolyte equilibrium in the intestines, resulting in watery diarrhoea (Zarei Ahmady et al. 2023). In this study, *estA* and *elt* genes, virulence factors of ETEC strains, were identified in 22.72% (5/22) and 27.27% (6/22) of *E. coli* isolates, respectively. Numerous publications have already disclosed that EPEC strains possess the *eaeA* gene (Younis et al. 2021). The *eaeA* gene was detected in 4% of *E. coli* strains in a prior investigation (Bean et al. 2004).

Lead, cadmium and arsenic are heavy metals capable of contaminating milk, thereby presenting significant health hazards. Lead serves as a powerful neurotoxin, especially detrimental to developing brains, resulting in developmental delays, learning disabilities and behavioural issues (Farahmandkia et al. 2025). Cadmium can cause damage to the kidneys and liver and is associated with an increased chance of specific cancers. Arsenic exposure can elevate the risk of multiple cancers, including those of the bladder, lung and skin, while also adversely affecting the cardiovascular and nervous systems (Norouzirad et al. 2018). In this study, the average concentration of heavy metals Cd, As and Pb was detected in the range of 0.0020–0.0053 ppm. Various European nations have documented disparate concentrations of Pb in milk (mg/L). Specifically, Slovenia recorded 0.05 mg/L, Spain 0.0018 mg/L, Austria 0.0065 mg/L, Italy 0.0013 mg/L, whereas Romania exhibited Pb levels ranging from 0.052 to 0.617 mg/L (Ali et al. 2023). Cadmium levels in milk vary across different countries. In Iran, the average level was 0.002 mg/L (Rahimi 2013). In Pakistan, the levels showed a wider range, from 0.001 to 0.053 mg/L (Ali et al. 2023). Cow's milk in Saudi Arabia had a cadmium level of 0.0047 mg/L (Farid et al. 2004). The low level of cadmium, lead and arsenic in this investigation demonstrates that CM samples contain these elements in slight levels. It is a source of pleasure to know that CM samples from various districts of Chaharmahal Bakhtiari province contain these metals at extremely low levels and are thus safe for consumers.

## 5 Conclusion

The detection of bacteria like *S. aureus* and *E. coli* in CM samples underscores the necessity of stringent hygiene and sanitation protocols during milking, processing and storage. Contamination may arise from multiple sources, including mastitis in the animals, unsanitary milking apparatus and inadequate storage conditions. This research reveals that the overall incidence of *E. coli* in 115 samples is 19.13%, whereas the prevalence of *S. aureus* is 23.47%. On the other hand, *sea*, *nuc*, *tst* and *pvl* genes were the most abundant genes found among *S. aureus* isolates. Moreover, *eaeA* and *bfp* genes, which are indicators of EPEC bacteria, were the most abundant in *E. coli* isolates. The identification of heavy elements, such as lead, cadmium and arsenic in some samples, highlights the need for monitoring environmental conditions that may affect milk quality. These results underscore the need of establishing stringent food safety protocols throughout the CM production chain to guarantee the safety and purity of this essential food source for consumers.

## Author Contributions

Elahe Yazdanian Ghahfarokhi and Amir Shakerian participated in the main idea of the experiment design. Reza Sharafati Chaleshtori and Ebrahim Rahimi participated in the design of the study and performed the statistical analysis.Amir Shakerian is responsible for the overall project and submission of article and correspondence. Elahe Yazdanian Ghahfarokhi and Ebrahim Rahimi are responsible for doing tests and editing the article.

## Consent

The authors have nothing to report.

## Conflicts of Interest

The authors declare no conflicts of interest.

## Data Availability

All data examined in this investigation are included in this manuscript for publication.
